# Lopinavir/ritonavir monotherapy in clinical practice

**DOI:** 10.1186/1758-2652-13-S4-P50

**Published:** 2010-11-08

**Authors:** A Caso, E Valencia, V Moreno, M Cervero, J Sanz, R Torres Perea

**Affiliations:** 1 Hospital Principe de Asturias, Internal Medicine, Alcalá de Henares (Madrid), Spain; 2 Hospital Carlos III, Infectious Diseases, Madrid, Spain; 3 Hospital Severo Ochoa, Internal Medicine, Leganés, Madrid, Spain

## Purpose of the study

To assess the usefulness of monotherapy with lopinavir/ritonavir (LPV/r) as an option for antiretroviral treatment in clinical practice.

## Methods

Seventy-seven subjects (56 men, median age 44.5 years) with HIV-1 RNA <50 copies/mL for at least 6 months, were switched to LPV/r as single antiretroviral agent. Reason for changes were simplification strategy (36.4%) or toxicity (63.6%) either mitochondrial toxicity (55.8%) or other (7.8%). Treatment with LPV/r was maintained for at least 3 months.

## Summary of results

The average time from HIV-1 diagnosis to starting HAART was 54 months. Patients had received a median of 7 antiretroviral drugs (range 3-14). The previous antiretroviral regimen included LPV/r in 55 (71.4%) patients. After a mean (±SD) follow-up of 25 (±16) months (median 22 months), viral load remained undetectable in 68 patients (90.7%) (9 of them after reintroduction of triple therapy for reasons other than virological failure), and virological failure was detected in 9 (11.7%), due to poor adherence in 7 (77.8%). The median time of undetectable viral load prior to initiating LPV/r monotherapy was 36 months. The mean CD4+ T cell count at the time of beginning LPV/r was 518.9 cells/mm^3^ (range 33−1433) and the end of follow-up 634.5 cells/mm3 (range 99−1547), with an increase of 115.6 cells/mm^3^. In 8 patients, 13 blips were detected (viral loads > 50 copies/mL and < 500 copies/mL), which did not warrant a change in therapy. Differences between patients with and without virological failure during LPV/r monotherapy included: older age at HIV-1 diagnosis (40.2 vs 30.7 years, P < 0.049), time of undetectable viral load prior to starting monotherapy (29 vs 45 months, P = 0.05), and CDC category C (77.8% vs 42.6%, P = 0.074). On the other hand, there were no significant differences according to sex, risk group, previous failure to PIs( 9 patients), nadir CD4+ T cell count, or reasons to change to monotherapy. In the Cox regression analysis, age was independently associated with virological failure.

## Conclusions

LPV/r monotherapy has been an effective alternative in clinical practice either as a simplification strategy or in patients in which toxicity reduces the selection of antiretroviral drugs. Poor adherence and greater age were related to a higher rate of therapeutic failure.

